# Optimized breeding strategies for multiple trait integration: II. Process efficiency in event pyramiding and trait fixation

**DOI:** 10.1007/s11032-013-9937-6

**Published:** 2013-08-15

**Authors:** Ting Peng, Xiaochun Sun, Rita H. Mumm

**Affiliations:** 1 Department of Crop Sciences and the Illinois Plant Breeding Center, University of Illinois at Urbana-Champaign, 1102 S. Goodwin Ave., Urbana, IL 61801 USA; 2 Monsanto Company/Seminis Vegetable Seeds, Felda, FL USA; 3 Dow AgroSciences, Indianapolis, IN USA

**Keywords:** Computer simulation, Event pyramiding, Trait fixation, Seed chipping, Tissue sampling, Doubled haploid, Breeding strategy, Multiple trait integration

## Abstract

Multiple trait integration (MTI) is a multi-step process of converting an elite variety/hybrid for value-added traits (e.g. transgenic events) through backcross breeding. From a breeding standpoint, MTI involves four steps: single event introgression, event pyramiding, trait fixation, and version testing. This study explores the feasibility of marker-aided backcross conversion of a target maize hybrid for 15 transgenic events in the light of the overall goal of MTI of recovering equivalent performance in the finished hybrid conversion along with reliable expression of the value-added traits. Using the results to optimize single event introgression (Peng et al. Optimized breeding strategies for multiple trait integration: I. Minimizing linkage drag in single event introgression. Mol Breed, 2013) which produced single event conversions of recurrent parents (RPs) with ≤8 cM of residual non-recurrent parent (NRP) germplasm with ~1 cM of NRP germplasm in the 20 cM regions flanking the event, this study focused on optimizing process efficiency in the second and third steps in MTI: event pyramiding and trait fixation. Using computer simulation and probability theory, we aimed to (1) fit an optimal breeding strategy for pyramiding of eight events into the female RP and seven in the male RP, and (2) identify optimal breeding strategies for trait fixation to create a ‘finished’ conversion of each RP homozygous for all events. In addition, next-generation seed needs were taken into account for a practical approach to process efficiency. Building on work by Ishii and Yonezawa (Optimization of the marker-based procedures for pyramiding genes from multiple donor lines: I. Schedule of crossing between the donor lines. Crop Sci 47:537–546, 2007a), a symmetric crossing schedule for event pyramiding was devised for stacking eight (seven) events in a given RP. Options for trait fixation breeding strategies considered selfing and doubled haploid approaches to achieve homozygosity as well as seed chipping and tissue sampling approaches to facilitate genotyping. With selfing approaches, two generations of selfing rather than one for trait fixation (i.e. ‘F2 enrichment’ as per Bonnett et al. in Strategies for efficient implementation of molecular markers in wheat breeding. Mol Breed 15:75–85, 2005) were utilized to eliminate bottlenecking due to extremely low frequencies of desired genotypes in the population. The efficiency indicators such as total number of plants grown across generations, total number of marker data points, total number of generations, number of seeds sampled by seed chipping, number of plants requiring tissue sampling, and number of pollinations (i.e. selfing and crossing) were considered in comparisons of breeding strategies. A breeding strategy involving seed chipping and a two-generation selfing approach (SC + SELF) was determined to be the most efficient breeding strategy in terms of time to market and resource requirements. Doubled haploidy may have limited utility in trait fixation for MTI under the defined breeding scenario. This outcome paves the way for optimizing the last step in the MTI process, version testing, which involves hybridization of female and male RP conversions to create versions of the converted hybrid for performance evaluation and possible commercial release.

## Introduction

Biotechnology has become an important component in the development of new and improved cultivars (Moose and Mumm 2008). The array of value-added traits created through the use of genetic modification has been expanding since genetically modified (GM) traits debuted in the mid-1990s, with events either commercialized or in development for herbicide tolerances, insect resistances, drought tolerance, nitrogen use efficiency, yield enhancement, grain composition modification (amino acid composition, protein content, and oil composition), disease resistances, grain processing enhancements (phytase for animal feed and amylase for corn ethanol), and other useful traits (Information Systems for Biotechnology 2012) which may be helpful to close the yield gap (Que et al. 2010). Furthermore, GM traits have been rapidly adopted by farmers worldwide as economic and environmental benefits have been realized (Brookes and Barfoot 2012). This has fueled the trend to include more and more GM traits in new cultivars, a practice referred to as ‘stacking’. It is predicted that as many as 15–20 value-added traits may be stacked in new maize cultivars by 2030 (Que et al. 2010; Fraley 2012).

The process of converting a target cultivar for multiple traits (or transgenic events), i.e. multiple trait introgression (MTI), has been widely practiced in maize breeding. This process usually consists of four steps: single event introgression, event pyramiding, trait fixation, and version testing (performance testing of various versions of a given target hybrid conversion). The overall aim of MTI is to recover at least one version of the converted target hybrid with equivalent performance to the unconverted target hybrid and stable expression of all the value-added traits. The probability of success depends greatly on the amount of non-recurrent parent (NRP) germplasm from event donors that can be eliminated in the MTI process as inbred parents of the target hybrid are converted. Minimization of NRP germplasm in close proximity to the chromosomal location of the event insertion (i.e. linkage drag) is particularly critical, especially given use of a non-elite transformation line, e.g. Hi-II (Armstrong et al. 1991); somaclonal variation resulting from tissue culture during the transformation process; and use of a donor parent from the opposite heterotic group (e.g. donor from the female heterotic group to convert a line from the male heterotic group). The latter is particularly pertinent to the development of new stacked cultivars since every event originates from a single T0 plant (generation arising directly from the transformation/regeneration process). As such, success demands an integrated approach across the four steps of MTI, yet requires specific breeding objectives to be realized at each step along with operational efficiency. Typically, molecular markers are utilized in MTI for efficiency, speed, and improved probability of recovering equivalent performance in the converted hybrid relative to the unconverted target hybrid.

We have approached MTI with the overarching aim of identifying an optimized breeding strategy to convert a target maize hybrid for 15 transgenic events and capture yield performance equivalency within a strict range, i.e. 3 %. We developed a realistic breeding scenario that might be encountered in the seed industry which assumes that (1) the transformation line is considered to be related to the female side of the heterotic pattern, and (2) some events are required on the male side of the target hybrid; therefore, to balance out the number of events for introgression into each parent, eight events will be introgressed in the female RP and seven events into the male RP; (3) all events are new so conversions for each event are required; (4) events to be combined in a given RP are not linked genetically; (5) residual NRP germplasm in the 20 cM region flanking the event insertion (FR NRP) will be essentially unalterable after the single event introgression step is completed and event pyramiding begins; and (6) 120 cM of NRP germplasm (i.e. ~96.66 % RP recovery given a genome size of 1,788 cM) represents a threshold for residual NRP germplasm consistent with a high probability of recapturing target hybrid performance (Sun 2012). With 15 events overall, this requires ≤8 cM Total NRP in each single event conversion. Furthermore, because we assumed that FR NRP will be unalterable after single event introgression is completed and event pyramiding begins, we arbitrarily designated the threshold for FR NRP for each single event introgression to be ~1 cM.

Using computer simulation, an optimal breeding strategy for the first step in MTI to accomplish breeding objectives specific for single event introgression was identified (Peng et al. 2013). This strategy involved a selection scheme featuring five backcross generations of marker-aided backcrossing, with BC1 through BC3 selected for the event of interest and minimal linkage drag at population sizes of 600, and BC4 and BC5 selected for the event of interest and recovery of the RP germplasm across the genome at population sizes of 400, and selection intensity of 0.01 for all generations. Thus, through computer simulation, we saw that it is indeed possible to recover single event RP conversions with ≤8 cM residual NRP germplasm with ~1 cM in the region flanking the event insertion.

**Fig. 1:**
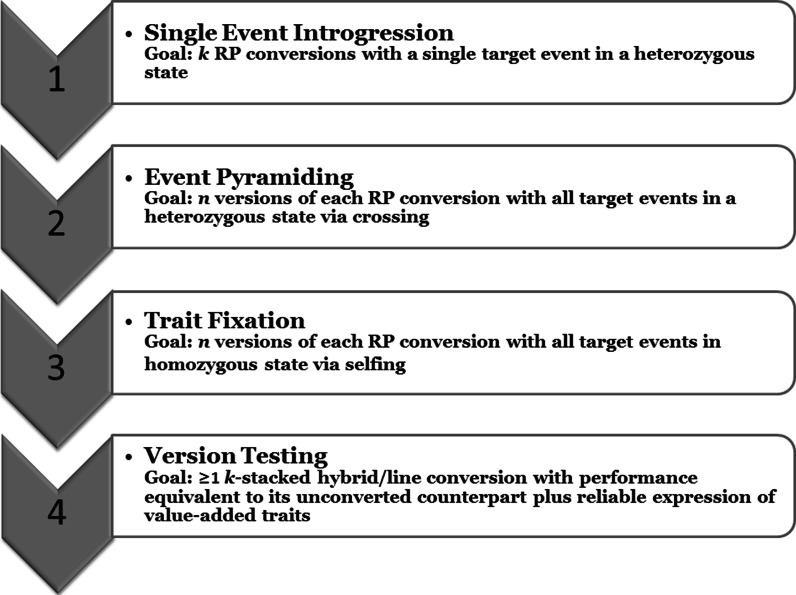
Steps and associated goals to achieve desired outcomes in multiple trait introgression (MTI) to produce a hybrid conversion for *k* events

Now, with this study, we turned our attention to optimization of the next two steps in MTI, event pyramiding and trait fixation, using the single event introgression products as the starting materials (Fig. 1). The breeding goal for event pyramiding is to assemble all the specified events in the target RP by crossing single event conversions to create stacked versions of each RP with all events in a heterozygous state. Then, for trait fixation, the breeding goal is to recover at least one line which is homozygous for all event loci to ensure stable expression of value-added traits. Optimized breeding strategies for these steps would highlight process efficiency and expediency.

Several studies have considered optimal approaches for event pyramiding. Servin et al. (2004) pointed out that as the number of target genes to be pyramided increases, the number of ways to arrange the crossing schedule increases dramatically; they provided an algorithm to calculate the optimal crossing schedule for a given number of target genes to be pyramided. Ishii and Yonezawa (2007b) concluded that the crossing schedule should be as symmetrical as possible, assuming the crossing schedule features parallel streams to ultimately assemble all events in the target RP. Guidelines to deal with linked target genes (or events) were provided in several studies (Servin et al. 2004; Ishii and Yonezawa 2007a; Wang et al. 2007; Ye and Smith 2010). In this study, single event introgression was conducted prior to the event pyramiding step. Furthermore, we assumed no linkage between events (i.e. for each RP, events to be stacked are located on different chromosomes).

With trait fixation, the goal of recovering one or more families homozygous for all events is relatively simple when one or two events are involved, typically requiring only one generation of self-pollination with reasonable population size to achieve the desired outcome. However, once the number of target events exceeds five, the frequency of individuals with all target event loci in a homozygous state within one selfing generation is extremely low. For example, the estimated frequency of individuals with eight events in a homozygous state equals (0.25)^8^ = 0.00001526. With such a low probability, the minimal number of families required to find one or more with the desired genotype is 301,803 (as per Mainland 1951), which is beyond the population size that could realistically be accommodated resource-wise in an actual breeding program. To add to the complexity, usually multiple versions of the stacked RP conversion are created in order to ensure recovery of one or more versions with equivalent performance to the unconverted target hybrid. Thus, given the need for *n* versions of the RP, each with a minuscule probability, the total minimal population would be even larger.

Bonnett et al. (2005) proposed an ‘F2 enrichment’ strategy to counter the demand for large population sizes due to low frequency of the desired genotype, suggesting a two-generation approach to fix all the targeted trait (or event) loci. With this approach, in the first selfing generation (i.e. S1), genotypes with all target events either in a heterozygous or homozygous state (i.e. AA and Aa) are selected with expected probability of 0.75 per locus, and in the second selfing generation, genotypes with all target events in a homozygous state are recovered with expected probability of 0.5 per locus. For example, using this ‘F2 enrichment’ strategy, if the breeding goal is to fix eight target events loci, the frequency of the desired genotype in the first generation (S1) is (0.75)^8^ = 0.1001129 and in the second generation (S2) is (0.5)^8^ = 0.00390625. Thus, the minimal population size to find one or more desired genotypes in the first generation is only 44 and in the second generation is only 1,177 (as per Mainland 1951), which dramatically decreases the total population size necessary to achieve the breeding goal from 301,803 to 1,221; however, the trade-off is an extra generation. Wang et al. (2007) confirmed the superiority of this approach with their simulation study. Likewise, Ishii and Yonezawa (2007b) compared four different selection strategies for trait fixation with multiple target genes in a heterozygous state using computer simulation, some involving doubled haploids and others involving intercrosses among ‘most complete’ selections when the desired genotype was not recovered. However, Ishii and Yonezawa (2007b) concluded that recurrent selection (crossing among selections) is not necessary if the total number of target events is less than ten (which includes the case involving fixation of eight or seven targeted trait loci in our breeding scenario).

In light of the need for an integrated approach across MTI to achieve success in the conversion of a target corn hybrid for 15 transgenic events, the objectives of this work were to (1) fit an optimal breeding strategy for pyramiding eight events in the female RP (and seven in the male RP) based on published work by Ishii and Yonezawa, and (2) evaluate optimal breeding strategies for trait fixation to create a ‘finished’ conversion of each RP homozygous for all events, focusing on process efficiency. The latter considered selfing and doubled haploid approaches to achieve homozygosity as well as seed chipping and tissue sampling approaches to facilitate genotyping. Efficiency indicators such as total number of individuals (plants grown) across generations (NPG), total number of marker data points (MDP), total number of generations (GEN), number of seeds sampled by seed chipping (NSC), number of plants requiring tissue sampling (NTS), and number of pollinations (NP) (i.e. selfing and crossing) were considered in evaluating breeding strategies. Computer simulation and probability theory were used to explore the myriad of potential options based on numerical estimations for these efficiency indicators. We also considered next-generation seed needs, which is a very practical aspect of the process, yet key to optimal efficiency.

## Materials and methods

### Genetic simulation

Computer simulations in this study were conducted using R statistical software. Models of the genome and the MTI process were developed as outlined in Peng et al. (2013). The genome model for simulation was constructed according to the published maize ISU–IBM genetic map, with a total of 1,788 cM (Fu et al. 2006). With the focus of this study on event pyramiding and trait fixation, marker tracking involved detection of each event and distinction between a heterozygous and homozygous state. To facilitate selection for each event, a single marker serving as a perfect marker for the event was simulated. The process model was used to create progeny genotypes produced through crossing, backcrossing, selfing, or doubled haploidy and accounted for the results of selection in each generation.

**Fig. 2:**
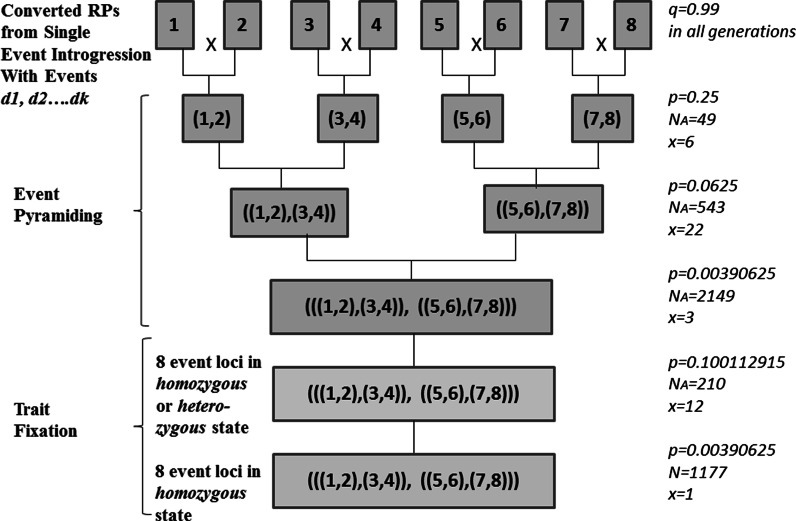
Using the SC + SELF breeding strategy as an example, the crossing schedule for event pyramiding and trait fixation is shown, featuring for each generation: the frequencies of the desired genotype (*p*), required population size (*N)* adjusted for seed needs in the next generation (*N*_A_; as per Eq. 1), and the number of selected individuals (*x*; also adjusted for seed needs in the next generation), assuming a 99 % success rate (*q*). The generational goals for trait fixation are specified; for event pyramiding, the goal each generation is to recover specified events in a heterozygous state

Building on work by Ishii and Yonezawa (2007a), a symmetrical crossing schedule for event pyramiding was devised for stacking eight events in a target RP (Fig. 2). This schedule features the conversion of the female parent of the target hybrid and, with minor adjustments, can also be used to demonstrate the stacking of seven events in male RP. The single event conversions of each RP produced according to the method proposed by Peng et al. (2013) served as the starting point. For trait fixation, six breeding strategies for recovering multiple families of a version of the target RP fixed for the eight or seven events were compared based on variations of self-pollination (SELF) or use of doubled haploidy (DH) as well as seed chipping (SC) or tissue sampling (TS). Seed chipping technology facilitates automated collection of plant tissue from a single seed in a non-destructive fashion, from which DNA will be extracted for marker genotyping (e.g. http://www.monsanto.com/products/Pages/breeding.aspx). This method of tissue collection is currently used not only with corn, but also with a wide array of grain and vegetable crops (Monsanto 2012). In modern plant breeding, DH breeding technology shows great advantage in producing ‘instant inbreds’, that is, fully homozygous lines in only 1–2 generations. It is commonly used in the seed industry to accelerate line development (Gallais and Bordes 2007; Choe et al. 2012) and has been implicated as a potential advantage in MTI, although it is not clear that it is currently being used for this purpose. With selfing approaches, the ‘F2 enrichment’ strategy proposed by Bonnett et al. (2005) was included in the process model to overcome the bottleneck represented with only one generation of selfing and extremely low frequency of desired individuals mandating huge population size.

**Fig. 3:**
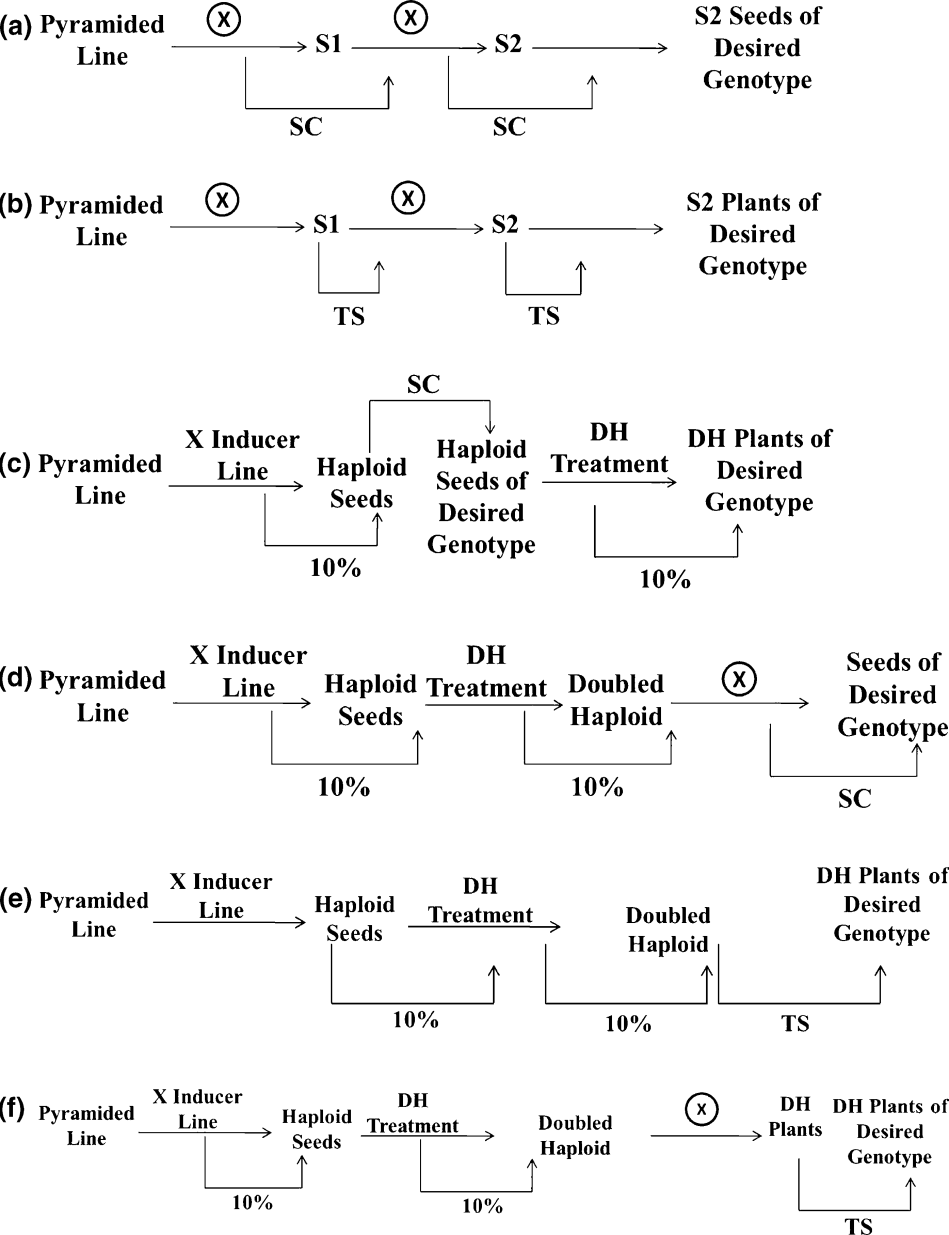
Descriptions of the six breeding strategies for trait fixation involving variations of self-pollination (SELF) versus use of doubled haploid (DH), and seed chipping (SC) versus tissue sampling (TS) to collect material for genotypic analysis. **a** Selfing with seed chipping (SC + SELF). **b** Selfing with tissue sampling (TS + SELF). **c** Doubled haploidy with seed chipping of haploid seeds (SC + DH-I). **d** Doubled haploidy with seed chipping of seeds from DH plants (SC + DH-II). **e** Doubled haploidy with tissue sampling of DH plants (TS + DH-I). **f** Doubled haploidy with tissue sampling of S1 individuals (TS + DH-II)

The six breeding strategies evaluated in this study comprised SC + SELF, TS + SELF, SC + DH-I, SC + DH-II, TS + DH-I, and TS + DH-II, which are depicted in detail in Fig. 3. SC + SELF is a breeding strategy involving two generations of selfing incorporating the ‘F2 enrichment’ approach (Bonnett et al. 2005) and utilizing SC for tissue collection (Fig. 3a). TS + SELF is a breeding strategy involving two generations of selfing incorporating the ‘F2 enrichment’ approach (Bonnett et al. 2005) and utilizing TS for tissue collection (Fig. 3b). SC + DH-I involves crosses between the event pyramiding selections and a haploid inducer in order to generate haploid seeds (Fig. 3c). The resulting haploid seeds are anticipated at a 10 % frequency in the seed bulk. SC genotyping will be applied to the identified haploid seeds in order to detect those with the desired genotype (i.e. all target events present). Next, selected haploid seeds will be germinated and treated with a chromosome doubling agent in order to recover DH plants. This doubling treatment has an estimated success rate of 10 %. SC + DH-II strategy differs from SC + DH-I in the generation for screening individual seeds for the desired genotype (Fig. 3d). With SC + DH-II, SC (and genotyping) is conducted after haploid plants are doubled and selfed to produce seed. In contrast, the TS approaches can be implemented only after DH plants are produced. With TS + DH-I, TS is implemented as soon as successfully doubled haploid plants are identified (Fig. 3e), whereas with TS + DH-II, TS and genotyping are conducted after successfully doubled haploid plants are self-pollinated to produce the next generation of seed (Fig. 3f).

The frequencies of the specified genotypes in the population were calculated according to Mendelian genetic principles for a diploid genome with bi-allelic loci stipulating the presence or absence of an event. Thus, the expected frequency of individuals with *n* target events in a heterozygous state is 0.5^*n*^ assuming no genetic linkage between any target events. In the trait fixation step, to employ the ‘F2 enrichment’ strategy in a two-generation selfing scheme according to Bonnett et al. (2005), in the first generation the frequency of individuals with *n* target events in either a heterozygous or homozygous state was 0.75^*n*^ and in the second generation, the frequency of individuals with *n* target events in a homozygous state was 0.5^*n*^. With DH, the frequency of haploid seeds from the cross with the inducer line as well as the probability of fertile diploid individuals resulting from successfully doubling chromosomal content with the application of a doubling agent was set to 0.10, in keeping with reports from Choe et al. (2012).

The minimum population size required in a given generation in keeping with a specified genotypic frequency and probability of success was computed in R based on the binomial distribution (Sedcole 1977):

1 $$ \mathop \sum \limits_{i = x}^{N} \left( \frac{N}{i} \right)q^{i} (1 - q)^{N - i} \ge p $$

where *N* refers to the minimal population size, *x* is the number of recovered individuals with the desired genotype, *p* is the frequency of the desired genotype in the population, and *q* is the probability of achieving the breeding goal.

The special case involving *x* = 1 is consistent with the goal of recovering at least one individual (e.g. one or more seed/plant/family) and the following simplified version of Eq. 1 by Mainland (1951) can be utilized:

2 $$ N \ge { \ln }(1 - q)/{ \ln }(1 - p) $$

However, in real life, recovery of more than one individual is typically desired to manage risks (e.g. germination failure) and is often required to meet seed needs for the next generation.

The breeding goal at the last generation in trait fixation aims to recover one or more families with all target event loci in a homozygous state in the RP. The probability of achieving the desired outcome was computed for each generation and used to estimate the minimum population size (*N*) needed to achieve the specified goal in each breeding step. The minimum population size was later adjusted upward (*N*_A_) to take the seed needs for next generation into consideration. In calculating estimates of *N*_A_, we assumed that an inbred plant produced 100 seeds on a single ear through selfing and that a DH plant produced 50 seeds.

### Comparison criteria

The six breeding strategies for trait fixation of eight target events were compared based on recovery of one or more families (i.e. one seed with SC and one plant for TS). Due to differences in the developmental stage in which tissue collection is performed, the desired genotypes being identified from the SC genotyping methods were seeds while the desired genotypes being identified from the TS genotyping methods were plants. We also assumed that the genotyping results were available before pollination for the strategies involving TS. Moreover, we defined one generation as the interval from harvested seed to plant maturity/harvest of the plant resulting from that seed. For example, S1 plants bearing S2 seed were not considered to be advanced to the next generation until S2 seed was harvested. However, selections based on S2 seed through SC were considered a half generation ahead of S2 plants resulting from S2 seed that had been planted and germinated as in TS.

Several criteria were considered to compare the efficiency of each breeding strategy. The comparison parameters include NPG for estimating the field resource requirements; MDP for estimating the genotyping demands; GEN for estimating the time requirement; NSC and NTS for estimating capital investment and labor requirements; and NP for estimating the nursery requirements. These statistics can then be used by readers to estimate resource costs associated with specific breeding strategies based on resource charges specific to their organization.

## Results and discussions

### Population size

Breeding strategy options were outlined based on a 99 % probability of achieving breeding objectives in each generation and recovering one or more families of the stacked RP conversion (i.e. one or more seeds for breeding strategies involving seed chipping; one or more S1 plants or S2 families for breeding strategies involving tissue sampling) homozygous for all events at the close of trait fixation. For each generation in event pyramiding and trait fixation, the probability of achieving the desired outcome was computed (Table 1). The probabilities of success for each generation ranged from 0.25 (to pyramid two events) to minute likelihoods of 0.00390625 (e.g. to pyramid eight events and to recover all eight events in a homozygous state after selfing). The probability of success estimate was used to compute the minimum population size (*N*) needed to achieve the specified goal in each breeding step with each breeding strategy, which was later adjusted upward (*N*_A_) to take into consideration any additional seed needs to produce the necessary size of next generation. For example, in pyramiding two events by crossing single event RP conversions, the frequency of the double event heterozygotes among the progeny is 0.25 (Fig. 2). To recover one or more individuals of this type with 99 % probability, a minimum population size (*N*) of 16 is required (per Eqs. 1, 2). However, because 543 seeds are needed to produce the next generation to pyramid four events, six individuals of this genotype each producing 100 seeds apiece must be recovered rather than one. Therefore, the population size must be adjusted to accommodate this need; *N*_A_ is calculated as 49 (per Eq. 1) in keeping with a 99 % probability of recovering six or more double event heterozygote progeny from the cross of single RP conversions.

**Table 1:** Frequencies of desired genotypes in successive generations through event pyramiding and trait fixation in the conversion for eight events

	Breeding goal					
	Pyramid 2 events	Pyramid 4 events	Pyramid 8 events	F2 with 8 target events in heterozygous/homozygous state	F3 with 8 target events in homozygous state	Haploid or doubled haploid population with 8 target events
Desired genotype	Aa	Aa	Aa	AA/Aa	AA	A or AA
Formula	(0.5)^2^	(0.5)^4^	(0.5)^8^	(0.75)^8^	(0.5)^8^	(0.5)^8^
Probability	0.25	0.0625	0.00390625	0.100112915	0.00390625	0.00390625

The final generation of trait fixation is considered a success with recovery of one or more individuals homozygous for all eight events. Regardless of the breeding strategy, the need to increase the seed of the recovered family prior to version testing is pre-eminent since the outcome with all options considered a small number of seeds (e.g. 1–100 depending on the particular breeding strategy option).

### Event pyramiding breeding strategy

Event pyramiding was simulated using the single event conversions of the RP described by Peng et al. (2013) as starting materials (Fig. 2). Consistent with the breeding goal of integrating 15 transgenic events in the target hybrid, eight events were pyramided into the parent from the female heterotic group and seven other events were pyramided into the parent from the male heterotic group. Each of the RP conversions contained one of eight (seven) events with ≤8 cM Total NRP germplasm including ~1 cM in the 20 cM region of the genome flanking the event. Thus, event pyramiding was initiated with quality conversions with minimal linkage drag.

The breeding methodology for event pyramiding was adopted from Ishii and Yonezawa (2007a). With the goal for this step in MTI of creating a stacked version of the RP with all target events in a heterozygous state, a symmetrical structure was employed in the design of the event pyramiding crossing schedule (Fig. 2). To introgress eight events into female RP, a completely symmetrical crossing structure was used. To introgress seven events into the male RP, a combined crossing structure was used; a tandem structure was used in the first generation of crossing, followed by a symmetrical structure in later generations (not shown). No comparisons between crossing schedule options were necessary as Ishii and Yonezawa (2007a) had already established the efficiency of the symmetrical approach to this step in MTI in requiring the smallest total population size, and fewest GEN, MDP, and total NP. Nonetheless, to craft an overall breeding strategy for successful MTI, this step represents an important component of the overall breeding plan.

### Comparison of trait fixation breeding strategies

Six breeding strategies (Fig. 3) for trait fixation of eight events in a given RP were compared for NPG, MDP, GEN, NSC, NTS, and NP. All six breeding strategy options require only 1–2 generations, which is reasonable in industrial-scale breeding programs. Comparisons between the six breeding strategies facilitated evaluation of SC versus TS as the method of collecting materials for genotypic analysis; SELF approaches versus DH approaches; SC with haploid seeds versus SC with DH seeds; and TS in the same generation as DH plant screening versus TS one generation after DH plant screening.

Comparing SC with TS for collecting materials for genotyping, the SC option showed great advantages with both SELF and DH breeding strategies (Table 2). For example, comparing SC + SELF with TS + SELF, SC enables reduction of numbers of plants in the field since individual seed selections are made before planting; NPG was decreased more than 92-fold (1,390 vs 15). Furthermore, TS requires a significant number of NTS and therefore considerable human labor resources to accomplish. With the SELF approach, SC and TS options require the same MDPs. However, with DH, SC of haploid seeds requires substantially more marker data points (MDP = 24,624) than other breeding strategies as well as much larger total population size across generations (NPG = 6,368). SC with DH seeds requires the same total marker data points (MDP = 9,416) as TS + DH-I and TS + DH-II breeding strategies but much smaller total population size in the field (NPG = 562). In general, TS requires larger NPG than SC and significant human labor for tissue sampling and pollination needs; this is especially the case with TS + DH-I. Overall, SC provided tremendous advantages for trait fixation in MTI in terms of resource allocation, with both SELF and DH approaches. Furthermore, use of SC resulted in 0.5–1 fewer generations to realize the breeding goal compared to TS options. However, this may not translate to a meaningful advantage considering that sufficient seed must be produced with either method to proceed to the next step in MTI.

**Table 2:** Total number of plants grown across generations (NPG), number of marker data points (MDP), number of generations (GEN), total number of seeds sampled by seed chipping (NSC), total number of plants requiring tissue sampling (NTS), and total number of pollinations (NP) (i.e. selfing or crossing) associated with implementation of the six trait fixation breeding strategies for recovery of one or more families fixed for eight events

	*SC + SELF	TS + SELF	SC + DH-I	SC + DH-II	TS + DH-I	TS + DH-II
NPG	15	1,390	6,368	562	17,657	1,703
MDP	11,096	11,096	24,624	9,416	9,416	9,416
GEN	1.5	2	1	1.5	1	2
NSC	1,387	0	15,578	1,177	0	0
NTS	0	1,387	0	0	1,177	1,177
NP	15	213	3,215	444	15,099	444

An asterisk (*) identifies SC + SELF as the most efficient

Comparing the SELF and DH approaches, SELF proved more efficient in some respects than the DH breeding method under the defined breeding scenario (Table 2). Using SC, the SELF option requires only 15 plants in the field while the DH option requires many more (NPG = 6,368 for SC + DH-I and NPG = 562 for SC + DH-II). More than twice the MDP is needed with the SC + DH-I versus the SC + SELF. With SC + DH-II, the marker data point requirement is slightly lower than with SC + SELF (9,416 vs 11,096). However, the nursery demand (NP) would be still larger than with SC + SELF. Thus, benefit from DH is questionable under such a breeding scenario. Furthermore, the DH platform demands special knowledge and capital investment to develop and operate. Overall, the SC + SELF breeding strategy was determined to be more efficient than the SC + DH-I and SC + DH-II breeding strategies. If only TS is available, DH requires larger NPG than SELF methods, even though slightly smaller MDP (9,416 vs 11,096) and smaller NTS are needed. In addition, DH methods demand much larger nursery requirements (NP) than SELF options. In general, doubled haploidy may have limited utility in trait fixation for MTI under the defined breeding scenario. Comparing SC with haploid seeds (SC + DH-I) with SC with DH seeds (SC + DH-II), with the same total generation number (GEN), SC with haploid seeds requires more than 10 times NPG than SC with DH seeds (6,368 vs 562). SC + DH-I also results in much larger MDP, NSC, and NP than SC + DH-II. SC + DH-I shows a 0.5-generation advantage over SC + DH-II; however, this may not impact the timing of product release. Clearly, SC + DH-I incorporates two probabilities involving the desired genotype: the frequency of haploid seeds resulting from the cross to the inducer line (0.10) and the frequency of individuals containing all events [(0.5)^8^ = 0.00390625] into one step, thus leading to large NPG, MDP, NSC, and NP. In addition, one concern is whether seed chipping is workable with haploid seeds. If, for example, the seed chipping contributed to decreased germination, the efficiency of the DH system would be compromised.

Comparing TS in TS + DH-I with TS in TS + DH-II, TS + DH-I requires much larger NPG and NP than TS + DH-II to achieve the benefit of saving one breeding generation. If time is critical in the whole breeding program and TS genotyping is the only option, the TS + DH-I breeding strategy may be preferable despite the large NPG and NP requirements.

Overall, the SC + SELF trait fixation breeding strategy was determined to be the optimal breeding strategy to fix eight target event loci in terms of efficiency. It combines the SC advantage point (vs TS) and the benefits of SELF (vs DH). Although selections are identified in the seed stage, this does not necessarily translate to time savings in product development and release. It does, however, enable conditions promoting seed set to be maximized at or after planting the identified seed(s).

In this study, we considered use of various breeding technologies. However, with the information provided, individual programs can tailor a breeding strategy for trait fixation based on their unique situation with respect to technologies, facilities, and corporate objectives. Of course, our calculations are based on the reproduction rate of maize (i.e. the number of seeds being generated by one cross) and the success rates at various points in the DH system (i.e. the frequency of haploid seeds from the cross with the inducer line and the success rate for doubling haploid plants and restoring fertility). Thus, inferences pertaining to other plant species (e.g. soybean) or given other success rates for the DH platform may be different from those stated here.

## Conclusions

Within the context of converting a target hybrid for 15 transgenic events, a symmetrical crossing schedule for event pyramiding has been fitted and an optimal breeding strategy for trait fixation has been identified from among six options. SC + SELF, a breeding strategy involving two generations of self-pollination incorporating the ‘F2 enrichment’ approach (Bonnett et al. 2005) and utilizing SC for tissue collection, was determined to be the most efficient for trait fixation considering time (GEN) and resource requirements (MDP, NPG, NSC, NTS, and NP). Three generations are required for event pyramiding the eight (seven) events and an additional 1.5 generations were needed to implement SC + SELF for trait fixation. This means that, from single introgression to trait fixation, an optimal approach to converting a target hybrid for 15 events with no less that 96.66 % RP germplasm recovery (i.e. ≤120 cM residual NRP germplasm, according to Peng et al. 2013) incorporating eight events on the female side and seven on the male side involves a total of at least 11.5 generations (seven for single event introgression, three for event pyramiding, and 1.5 for trait fixation).

This outcome paves the way for optimizing the final step in the MTI process, version testing, which involves hybridization of female and male RP conversions to create versions of the converted hybrid for performance evaluation to assess yield equivalency with the unconverted hybrid. Creating multiple versions of each conversion is a common practice designed to minimize the risk of failure to recover the target hybrid field performance after effort and investment in MTI. Key to optimization of version testing is determining the minimal number of versions necessary to ensure a high probability of recapturing the target hybrid performance.

## Abbreviations

DH: Doubled haploid
FR NRP: Amount of non-recurrent parent germplasm in the 20 cM region flanking the transgenic event
GEN: Total number of generations
MDP: Total number of marker data points
MTI: Multiple trait integration
NP: Total number of pollinations (i.e. selfing or crossing)
NRP: Non-recurrent parent
NSC: Total number of seeds sampled by seed chipping
NPG: Total number of plants grown across generations
NTS: Total number of plants requiring tissue sampling
RP: Recurrent parent
SC: Seed chipping
TS: Tissue sampling
